# Supplementary material for: Decoding brain structure-function dynamics in health and in psychosis via an autoencoder
Source: Sci Rep. 2025 Nov 14;15:40052. doi: 10.1038/s41598-025-24232-z (PMC12618595; doi:10.1038/s41598-025-24232-z)
Supplement: Supplementary file 1 — Supplementary Information. [file 41598_2025_24232_MOESM1_ESM.pdf]

Supplementary Material for: Decoding Brain Structure-Function Dynamics in Health and in Psychosis Via an Autoencoder

Qing Cai[1,2], Hannah Thomas[1,3*], Vanessa Hyde[1,4], Pedro Luque Laguna[1], Carolyn B. McNabb[1], Krish D. Singh[1], Derek K. Jones[1], and Eirini Messaritaki[1]

[1]Cardiff University Brain Research Imaging Centre (CUBRIC), School of Psychology, Cardiff University, Cardiff, UK
[2]School of Artificial Intelligence, Tiangong University, Tianjin, China
[3]School of Medicine, Cardiff University, Cardiff, UK
[4]Cambridge University, Cambridge, UK
[*]Corresponding author: thomash66@cardiff.ac.uk

1 Data acquisition and Preprocessing

1.1 Sample

Multi-modal (MRI and MEG) data were collected from healthy participants via the Welsh Advanced Neuroimaging Database (WAND) study[1]. In this work, we use data from those participants that have good quality MRI and MEG data. Under these conditions, our sample consists of 126 healthy participants between the ages of 18 and 50 (73 female), 101 of whom were between the ages of 18 and 35 (55 female). The age distributions of the participants are shown in Fig. 1). The same data were collected from 5 people with psychosis between the ages of 18 and 35 (3 female). All psychosis participants were diagnosed at least 9 months prior to data collection.

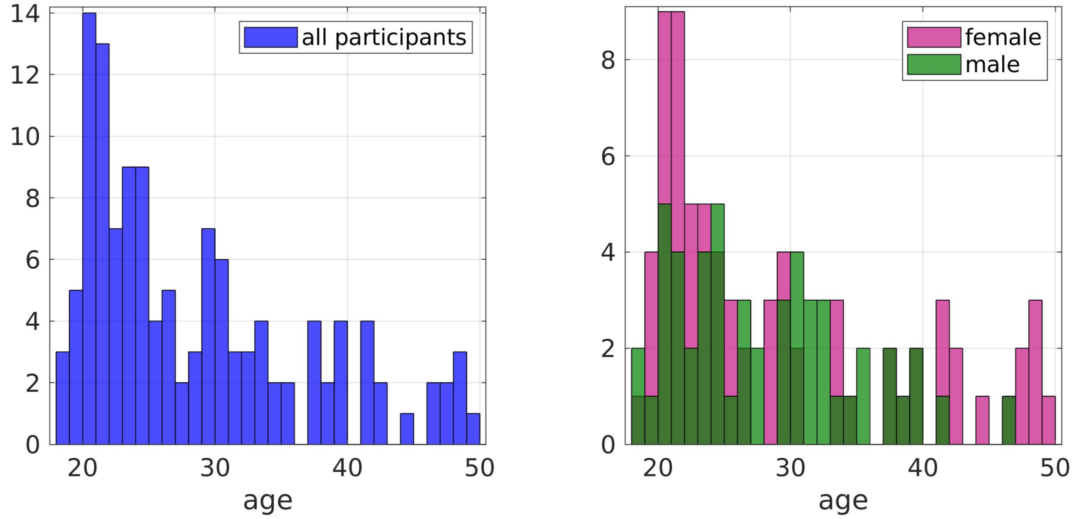

Figure S1: Age distributions of the healthy participants. The plot of the right panel breaks down the distribution by sex.

1.2 MRI

Data acquisition

All MRI data were acquired on a 3T Siemens Connectom scanner (300mT/m).

T1-weighted images were acquired using magnetization-prepared 180° radio-frequency pulses and rapid gradient-echo (MPRAGE) sequence, with repetition time (TR) 2300ms, echo time (TE) 2ms, field of view (FOV) 256 x 256 x 192 mm, matrix size 256 x 256 x 192, voxel size 1 x 1 x 1 mm, flip angle 9°, inversion time (TI) 857ms, in-plane acceleration (GRAPPA) factor 2 and phase-encoding direction anterior to posterior (A»P).

Multi-shell diffusion-weighted MRI data were acquired using protocols described recently for the Microstructural Image Compilation with Repeated Acquisitions (MICRA) dataset[2]. Data were acquired over 18 minutes using a single-shot spin-echo, echo-planar imaging sequence, in both anterior to posterior (A»P) and posterior to anterior (P»A) phase-encoding directions. A»P data comprised two shells of 20 diffusion encoding directions uniformly distributed (Jones et al., 1999) at b = 200 s/mm^2 and b = 500 s/mm^2, one shell of 30 directions at b = 1200 s/mm^2 and three shells of 61 directions each at b = 2400 s/mm^2, 4000s/mm^2 and 6000 s/mm^2. Additionally, two leading non-diffusion-weighted (b = 0 s/mm^2) images and 11 non-diffusion-weighted images were acquired, dispersed throughout (33rd volume and every 20nd volume thereafter). P»A data comprised two leading non-diffusion-weighted images, one shell of 30 directions at b = 1200 s/mm^2 and a final non-diffusion-weighted image. Data acquisition details for all b-values are as follows: TR=3000ms, TE=59ms, FOV = 220 x 200 mm in-plane, matrix size 110 x 110 x 66, voxel size 2 x 2 x 2 mm, with in-plane acceleration (GRAPPA) factor 2. The diffusion gradient duration and separation were 7ms and 24ms, respectively.

Data analysis

Data were analyzed on a CentOS Linux 7 cluster computing system.

Cortical reconstruction and volumetric segmentation of the T1-weighted images was performed with the FreeSurfer image analysis suite (`https://surfer.nmr.mgh.harvard.edu`). The technical details of the methods used by the FreeSurfer software have been described in other publications[3–15]. The Desikan-Killiany atlas[16] was used to identify the grey matter areas that form the nodes of the structural brain networks. The atlas provides 82 cortical and subcortical areas for the left and right cerebrum.

The diffusion-MRI data were corrected for thermal noise, signal drift, susceptibility distortions, motion and eddy-current distortions, gradient non-uniformity and Gibbs ringing artifacts using a combination of in-house pipelines, the FMRIB Software Library[17] and MRtrix3[18]. A brain mask was created using the Brain Extraction Tool[17] to exclude non-brain data. Diffusion MRI noise level estimation and denoising was performed using a Marchenko-Pastur principal component analysis (MP-PCA)-based approach[19,20]. Within-image intensity drift was corrected by fitting the diffusion-MRI data to temporally interspersed b0 images using in-house code in MATLAB R2017b (MathWorks Inc. Natick, Massachusetts, USA). Slicewise OutLIer Detection (SOLID)[21] was applied, with lower and upper thresholds of 3.5 and 10 respectively, using a modified z-score and a variance-based intensity metric. The susceptibility-induced off-resonance field was estimated from the b0 data collected in opposing phase-encoding directions using FSL's topup[22,23] and corrected, along with eddy-current induced distortions and participant movement using FSL's eddy tool[24]. In-house code was used to correct for gradient non-uniformity distortions in MATLAB 2017b (MathWorks Inc. Natick, Massachusetts, USA). Gibbs ringing correction was performed in MRtrix3[18] using the subvoxel-shifts method[25].

MRtrix3[18] was used to calculate the response function using the Dhollander algorithm[26,27], and the fiber orientation distributions using Multi-Shell-Multi-Tissue constrained spherical deconvolution[28], in order to perform anatomically-constrained tractography. The MRtrix function 5ttgen[23,29–32] was used to segment anatomical images into cortical grey matter (GM), subcortical grey matter, cerebrospinal fluid, white matter (WM) and abnormalities. Anatomical images were coregistered to the diffusion-weighted images using FSL[17,23,33–35]. The interface between grey and white matter was identified using function 5tt2gmwmi of MRtrix3[18,30]. Anatomically-constrained streamline tractography[18,30,36] was used to generate whole-brain tractograms with seeds in the GM-WM interface. Streamlines were of minimum/maximum length of 30/250mm, cutoff of 0.06, and maximum angle between successive steps of 50°. Twenty million streamlines were generated for each participant. The sift2 algorithm[37] was used to provide tractograms that have a density of reconstructed connections proportional to the fibre density within each voxel as estimated by the diffusion model. This ensures that the number of streamlines connecting two regions of grey matter provides an estimate of the cross-sectional area of the white matter axons connecting those regions, which is a biologically-relevant measure of structural connectivity. Each participant's tractogram was overlayed on the participant's fractional anisotropy map and visually inspected to ensure that the tractogram provided good coverage of the white matter and that no streamlines extended into unphysical regions.

The structural connectivity matrices representing the structural networks of the participants have the cortical and subcortical areas identified from the Desikan-Killiany atlas as the nodes, and the white matter tracts linking those areas as the edges (connections). To evaluate the impact that different white matter characteristics have on predicting electrophysiological resting-state FC, we used the following 5 structural measures as edge weights in the SC matrices.

1. Number of reconstructed streamlines (NS) connecting two cortical regions, calculated from the tractograms: SC matrices weighted with NS are good at predicting functional connectivity from both fMRI[38,39] and MEG[40].

2. Volume-normalized NS (NS/v): Because the absolute number of streamlines of a connection depends on the size of the connected brain areas (i.e., pairs of larger brain areas are connected by more streamlines because of their size rather than because of stronger connectivity) we divided the NS by the sum of the volumes of the connected brain areas.

3. Fractional anisotropy (FA) of the diffusion tensor, calculated by mapping the FA maps derived from the diffusion-weighted images to the streamlines in the tractogram[18]: The FA is related to the myelination and axonal characteristics of white matter tracts, and therefore is a good proxy for structural connectivity[38,40–43].

4. Inverse radial diffusivity (iRD) of the diffusion tensor, calculated by mapping the iRD maps derived from the diffusion-weighted images to the streamlines in the tractogram[18]: The iRD is related to the myelination of the white matter tracts, and has been used in studies in health and disease[40,44].

5. Total restricted signal fraction (FRt): derived from the CHARMED model[45], attributed to water within the intra-axonal space and therefore related to axonal characteristics of the WM tracts.

Our choice to investigate the FRt is motivated by the non-specificity of the FA and iRD to biological attributes of the brain's WM, such as myelination and axonal diameter. For example, a reduction in FA could reflect reduced neurite density, increased neurite orientation dispersion, both, or various other changes to tissue microstructure[46]. Radial diffusivity can be influenced by both myelination and axonal density of the white matter tracts, and therefore higher values can indicate lower myelination or axonal density[47–49].

With these 5 measures as edge weights (NS, NS/v, FA, iRD, FRt) we have 5 SC matrices for each participant. These 5 matrices all represent the participant's structural substrate that underlies FC, but each relies on a different aspect of the brain's microstructure to depict structural connectivity strength. The SC matrices were normalized so that the values for each SC matrix of each participant were in the range of 0 to 1.

The Euclidean distance (ED) between centers of the Desikan-Killiany brain areas was calculated for all participants. Specifically, MRtrix[18] was used to locate the coordinates of the center of each brain area, and in-house Matlab code was used to calculate the distances between those. Structural matrices were then calculated in which the values of structural connections were the Euclidean distances between the centres of the DK brain areas.

1.3 MEG

Data acquisition

Ten-minute whole-head MEG recordings were acquired in a 275-channel CTF radial gradiometer system, at a sampling rate of 1,200 Hz. Twenty-nine additional reference channels were recorded for noise cancellation purposes and the primary sensors were analyzed as synthetic third-order gradiometers[50]. Participants were seated upright in a magnetically shielded room with their head supported with a chin rest to minimize movement. They were asked to rest with their eyes open and to fixate on a central red point presented on a PROPixx LCD projector (Vpixx Technologies Inc). Horizontal and vertical electro-oculograms (EOG) were recorded to monitor eye blinks and eye movements. Recordings were also acquired while the participants performed tasks after the completion of the resting-state paradigm, but those recordings were not used in the analysis presented here.

Data analysis

To achieve MRI/MEG co-registration, fiduciary markers were placed at fixed distances from three anatomical landmarks identifiable in the participant's T1-weighted anatomical MRI scan, and their locations were manually marked in the MR image. Head localization was performed at the start and end of the MEG recording and continuously through the recording. The data were subsequently pre-processed in a manner similar to that described in previous work[51]. Specifically, all datasets were downsampled to 600 Hz, and filtered with a 1 Hz high-pass and a 150 Hz low-pass filter. The datasets were then segmented into 2-s epochs, and were visually inspected. Epochs exhibiting large head movements, muscle contractions, or other large artefacts were excluded from subsequent analysis.

The functional networks were constructed in a manner similar to that described in earlier work[51] but with some important differences. Specifically, the MEG sensor data were downsampled to 300 Hz, before being source localized using FieldTrip (RRID:SCR_004849) version 20190219[52]. The source-reconstruction grid for each participant was constructed using Freesurfer to segment the individual's T1 anatomical scan to yield the set of gray-matter voxels at a resolution of 2 mm isotropic. All these voxels were automatically labelled using the Desikan-Killiany parcellation[16] and matched to the MEG-coregistered MRI for the individual using FSL-FLIRT. All of these coregistered and parcellated gray-matter voxels were used for LCMV beamformer source-localisation using a single-shell forward model[53], where the covariance matrix was constructed in each of the four lower frequency bands: delta (1–4 Hz), theta (4–8 Hz), alpha (8–13 Hz), and beta (13–30 Hz). For each band, the beamformer weights were normalized using a vector norm[54]. Epochs were concatenated to generate a continuous dataset that was projected through these weights to yield a virtual-sensor time course for each voxel and then band passed to the above-mentioned frequency bands. For each of the 82 Desikan-Killiany regions, the virtual channel at the centroid of the region was chosen as a representative voxel.

The resulting 82 time series were orthogonalized using symmetric orthogonalization[55], to avoid spurious correlations. A Hilbert transform was used to obtain the oscillatory amplitude envelope. The data were subsequently despiked using a median

filter in order to remove artifactual temporal transients, downsampled to 1 Hz, and trimmed to avoid edge effects (removing the first two and the last three samples). To derive the amplitude-amplitude connectivity, amplitude correlations were calculated by correlating the downsampled Hilbert envelopes to each other, and the resultant correlation coefficients were converted to variance-normalized z-scores. This choice was motivated by the fact that such correlations have been shown to be one of the most robust and repeatable electrophysiological connectivity measures[56,57]. Specifically, these studies found that the oscillatory amplitude envelope correlation gave the most consistent connectivity, in contrast to other methods which lack repeatability over scans of the same subject. Given that we are comparing healthy participants to a patient population, it is important to have the most robust measure of functional connectivity possible.

Through this process we obtained 4 FC matrices for each participant.

2 Autoencoder Details

Based on the effectiveness of graph networks for representing brain topological structures, the encoder and decoder in this study are both selected to use graph network structures. Briefly, the encoder comprises two Graph Attention (GAT) layers. Each GAT layer utilizes 4 attention heads to capture diverse relational patterns. The input to the first layer is a node feature matrix derived from the SC matrix. The first GAT layer projects these features into a $64-$ dimensional space, and the second layer further compresses this into a $32-$ dimensional latent space representation for each node. The decoder then reconstructs the FC matrix from these latent representations. The model was trained using the Adam optimizer with a fixed learning rate of 10^{-3}, and we did not employ a learning rate schedule. To prevent overfitting and ensure the model did not continue training past the point of optimal performance, we implemented an early stopping mechanism: training was halted if the loss on the validation set did not improve for 20 consecutive epochs.

2.1 Encoder Structure

The graph attention mechanism is employed as the encoder layer to generate effective high-dimensional latent feature representations. By adding multiple layers of graph attention mechanisms to the encoder structure, the model's depth and learning capability are enhanced. Additionally, the representation learning of graph structure nodes produces richer node embeddings. To determine the relation between nodes and their neighbors, we use a self-attention mechanism that shares parameters between nodes. The input required by the encoder is the symmetrical adjacency matrix of the structural connectivity $A \in \mathbb{R}^{n\times n}$ and the set of node features $x = \{x_1, x_2, \ldots, x_n\}$, where $n = 82$ represents the number of nodes, i.e., the number of cortical regions partitioned in the DK brain atlas. The initial feature of node i is $h_i^0 = x_i \in \mathbb{R}^{1\times d}$. In the l-th layer of the encoder, the attention coefficient between node i and its neighbor node j is calculated as follows:

$$a_{ij}^l = \frac{\exp\left(\text{LeakyReLU}\left(\alpha^{l\top}\left[\mathbf{W}^l h_i^{l-1} || \mathbf{W}^l h_j^{l-1}\right]\right)\right)}{\sum_{k\in\mathcal{N}_i}\exp\left(\text{LeakyReLU}\left(\alpha^{l\top}\left[\mathbf{W}^l h_i^{l-1} || \mathbf{W}^l h_k^{l-1}\right]\right)\right)} \tag{1}$$

where $(\mathcal{N}_i)$ is a set of nodes connected to node i according to the adjacency matrix A, α denotes a learnable weight vector, $||$ represents the concatenation operation, $\mathbf{W}$ is a learnable linear transformation weight matrix, and LeakyReLU is the activation function. Further, the output representation of node i in the l-th layer of the encoder is as follows:

$$h_i^l = \sigma\left(\frac{1}{K}\sum_{k=1}^{K}\sum_{j\in\mathcal{N}_i} a_{ij}^l \mathbf{W}^l h_j^{l-1}\right) \tag{2}$$

After passing through L encoder layers, the output of the last layer is regarded as the final node embedding, i.e., $h_i = h_i^L, \forall i \in \{1, 2, \ldots, n\}$.

2.2 Decoder Structure

Contrary to the "compression" in the encoder, the decoder's role is to reconstruct the high-dimensional representations into the size of the functional connectivity matrix. We use a decoder with the same number of layers as the encoder, where each decoder layer reverses the process of its corresponding encoder layer, i.e., each decoder layer reconstructs the representation of the nodes by utilizing the representations of their neighboring nodes (according to their relevance). In the l-th decoder layer, the attention coefficient between node i and its neighbor node j is calculated as follows:

$$\hat{a}_{ij}^l = \frac{\exp\left(\text{LeakyReLU}\left(\hat{\alpha}^{l\top}\left[\hat{\mathbf{W}}^l \hat{h}_i^l || \hat{\mathbf{W}}^l \hat{h}_j^l\right]\right)\right)}{\sum_{k\in\mathcal{N}_i}\exp\left(\text{LeakyReLU}\left(\hat{\alpha}^{l\top}\left[\hat{\mathbf{W}}^l \hat{h}_i^l || \hat{\mathbf{W}}^l \hat{h}_k^l\right]\right)\right)} \tag{3}$$

Similar to Equation 1, $\hat{\mathbf{W}}$ and $\hat{\alpha}$ are learnable weight matrices.

Taking the output of the encoder as the input to the decoder, i.e., $\hat{h}_i^L = h_i^L, \forall i \in \{1,2,\ldots,N\}$, the representation of node i in the $(l-1)$-th layer of the decoder is reconstructed as follows:

$$\hat{h}_i^{l-1} = \sigma\left(\frac{1}{K}\sum_{k=1}^{K}\sum_{j\in\mathcal{N}_i}\hat{a}_{ij}^{l}\hat{\mathbf{W}}^{l}\hat{h}_j^{l}\right) \tag{4}$$

After passing through the same L layers of the decoder, the set of outputs of all nodes in the last layer is regarded as the reconstructed functional connectivity matrix, i.e., $\hat{y} = \{\hat{h}_1^0, \hat{h}_2^0, \ldots, \hat{h}_n^0\}$.

2.3 Objective Function

To achieve accurate predictions of functional connectivity from the structural connectivity matrices, the objective function of the neural network is designed to meet two requirements: 1) minimizing the prediction error between the oFC and the model-predicted pFC, which is achieved by calculating the mean squared error between oFC and pFC, 2) introducing a regularization term to ensure that the correlation (inter-pFC) between different individuals in the pFC is comparable to the correlation (inter-oFC) between different individuals in the oFC. This ensures that the neural network learns the mapping from SC to oFC while retaining individual differences, rather than predicting a population-average representation of functional connectivity for all individuals. Based on these two requirements, the objective function for the proposed overall structure is:

$$J = L(\theta) + \lambda(|\gamma - \phi(\theta)|) \tag{5}$$

where θ represents the parameters of the neural network to be learned, $L(\cdot)$ is the loss function, and λ is a regularization constant used in the regularization function $|\gamma - \phi(\cdot)|$, similar to the L1 norm. The mean squared error is used as the loss function, as shown below:

$$L = \frac{1}{n}\sum_{i=1}^{n}(y^i - \hat{y}^i)^2 \tag{6}$$

where n is the number of samples in a training batch, i represents a sample, y is the actual label (oFC), and $\hat{y}$ is the predicted output (pFC). The mean squared error ensures that the spatial correlation between SC and FC is approximately preserved during training.

The objective of the overall autoencoder structure is to predict the actual FC while also maintaining the inter-oFC differences between individuals. Therefore, a regularization term is added to the objective function, and the Pearson correlation coefficient r is introduced into the regularization function $|\gamma - \phi(\theta)|$:

$$r(a,b) = \frac{\sum(a-\bar{a})(b-\bar{b})}{\sqrt{\sum_{n-1}(a-\bar{a})^2}\sqrt{\sum(b-\bar{b})^2}} \tag{7}$$

$$\gamma = \frac{2}{n(n-1)}\sum_{i=1}^{n}\sum_{j=i+1}^{n} r\left(y^i, y^j\right) \tag{8}$$

$$\phi = \frac{2}{n(n-1)}\sum_{i=1}^{n-1}\sum_{j=i+1}^{n} r\left(\hat{y}^i, \hat{y}^j\right) \tag{9}$$

In the above equations, γ represents the inter-oFC correlation of the training dataset, which remains unchanged during training. It is noteworthy that γ varies for different datasets. In this case, ϕ represents the inter-pFC correlation, which is expected to be less than or equal to the inter-oFC correlation. Thus, the regularization function $\lambda(|\gamma - \phi(\theta)|$ ensures that the inter-individual differences in the pFC are comparable to the inter-individual differences in the oFC.

3 Performance Variability

To further assess the robustness of the model's outputs across subjects, we evaluated the stability of the correlation-based features over 5-fold cross-validation. Each fold contained 26, 25, 25, 25, and 25 subjects, respectively. For each feature matrix (NS, NS/v, FA, iRD, FRt), we computed the average value across subjects within each fold, and then measured the cross-fold standard deviation and 95% confidence intervals. The results are summarized in Table S1 below. Notably: the alpha and beta bands consistently show high stability, with standard deviations <0.02 and CI widths <0.04 across all feature types. The delta and theta bands show higher variability.

SC metric	FC frequency band	Mean	SD	95% CI	Fold Means
NS	delta	0.13	0.12	[-0.01, 0.28]	0.12, 0.26, 0.25, 0.02, 0.02
	theta	0.50	0.02	[0.48, 0.53]	0.53, 0.50, 0.48, 0.49, 0.53
	alpha	0.83	0.01	[0.81, 0.85]	0.84, 0.82, 0.85, 0.82, 0.84
	beta	0.85	0.01	[0.84, 0.87]	0.84, 0.88, 0.86, 0.85, 0.85
NS/v	delta	0.09	0.16	[-0.11, 0.29]	0.38, 0.02, 0.01, 0.03, 0.03
	theta	0.48	0.03	[0.45, 0.52]	0.50, 0.51, 0.45, 0.46, 0.50
	alpha	0.84	0.02	[0.81, 0.86]	0.86, 0.82, 0.85, 0.82, 0.84
	beta	0.85	0.01	[0.84, 0.87]	0.85, 0.86, 0.88, 0.84, 0.85
FA	delta	0.34	0.17	[0.12, 0.55]	0.51, 0.53, 0.25, 0.19, 0.20
	theta	0.49	0.18	[0.27, 0.72]	0.58, 0.60, 0.56, 0.57, 0.17
	alpha	0.86	0.01	[0.84, 0.87]	0.85, 0.86, 0.87, 0.84, 0.87
	beta	0.86	0.01	[0.85, 0.87]	0.86, 0.86, 0.87, 0.85, 0.86
iRD	delta	0.42	0.04	[0.37, 0.48]	0.47, 0.46, 0.43, 0.38, 0.39
	theta	0.51	0.06	[0.44, 0.58]	0.51, 0.56, 0.57, 0.44, 0.47
	alpha	0.86	0.01	[0.85, 0.87]	0.86, 0.86, 0.87, 0.84, 0.87
	beta	0.86	0.01	[0.85, 0.87]	0.88, 0.86, 0.87, 0.86, 0.86
FRt	delta	0.50	0.01	[0.48, 0.52]	0.48, 0.50, 0.52, 0.49, 0.51
	theta	0.48	0.14	[0.31, 0.66]	0.59, 0.57, 0.59, 0.35, 0.31
	alpha	0.84	0.03	[0.81, 0.87]	0.85, 0.86, 0.87, 0.80, 0.82
	beta	0.86	0.01	[0.84, 0.87]	0.85, 0.86, 0.87, 0.85, 0.85

Table S1 : Performance variability measures.

4 Cohen's d

In this section, we give the Cohen's d values for the comparisons of the performance of the two models between healthy and psychosis participants, and for the comparison of the two models for the psychosis participants.

	NS	Ns/v	FA	iRD	FRt
delta	1.1	1.4	13.6	10.3	9.1
theta	5.2	4.2	0.8	5.4	5.3
alpha	5.5	5.4	6.2	5.8	5.6
beta	4.5	5.6	5.5	5.4	5.6

Table S2 : Cohen's d for the comparison between healthy and psychosis participants when the GMHA-AE model is used for FC prediction.

	NS	Ns/v	FA	iRD	FRt
delta	-7.1	-6.2	-3.9	-4.0	-5.8
theta	-0.4	-0.8	0.3	0.3	-0.2
alpha	0.6	0.3	1.9	1.3	1.0
beta	0.8	0.7	1.9	2.1	2.1

Table S3 : Cohen's d for the comparison between healthy and psychosis participants when the analytical model is used for FC prediction.

	NS	Ns/v	FA	iRD	FRt
delta	16.9	10.0	5.9	5.9	8.2
theta	4.7	2.7	4.4	4.5	4.8
alpha	-2.9	-2.9	-2.6	-2.7	-1.9
beta	-3.6	-3.6	-6.5	-4.8	-2.7

Table S4 : Cohen's d for the comparison between the GMHA-AE model and the analytical model used for FC prediction in psychosis participants.

References

1. McNabb, C. *et al.* The welsh advanced neuroimaging database: an open-source state-of-the-art resource for brain research. *Proc. Annu. Meet. ISMRM* **241** (2024).
2. Koller, K. *et al.* Micra: Microstructural image compilation with repeated acquisitions. *NeuroImage* **225**, 117406 (2021).
3. Dale, A. M., Fischl, B. & Sereno, M. I. Cortical surface-based analysis: I segmentation and surface reconstruction. *NeuroImage* **9**, 179–194, DOI: 10.1006/nimg.1998.0395 (1999).
4. Dale, A. M. & Sereno, M. I. Improved localizadon of cortical activity by combining eeg and meg with mri cortical surface reconstruction: A linear approach. *J. Cogn. Neurosci.* **5**, 162–176, DOI: 10.1162/jocn.1993.5.2.162 (1993).
5. Fischl, B., Liu, A. & Dale, A. Automated manifold surgery: constructing geometrically accurate and topologically correct models of the human cerebral cortex. *IEEE Transactions on Med. Imaging* **20**, 70–80, DOI: 10.1109/42.906426 (2001).
6. Fischl, B. *et al.* Whole brain segmentation. *Neuron* **33**, 341–355, DOI: 10.1016/S0896-6273(02)00569-X (2002).
7. Fischl, B. Automatically parcellating the human cerebral cortex. *Cereb. Cortex* **14**, 11–22, DOI: 10.1093/cercor/bhg087 (2004).
8. Fischl, B. *et al.* Sequence-independent segmentation of magnetic resonance images. *NeuroImage* **23**, S69–S84, DOI: 10.1016/j.neuroimage.2004.07.016 (2004).
9. Fischl, B., Sereno, M. I. & Dale, A. M. Cortical surface-based analysis. *NeuroImage* **9**, 195–207, DOI: 10.1006/nimg.1998.0396 (1999).
10. Fischl, B., Sereno, M. I., Tootell, R. B. & Dale, A. M. High-resolution intersubject averaging and a coordinate system for the cortical surface. *Hum. Brain Mapp.* **8**, 272–284 (1999).
11. Fischl, B. & Dale, A. M. Measuring the thickness of the human cerebral cortex from magnetic resonance images. *Proc. Natl. Acad. Sci.* **97**, 11050–11055, DOI: 10.1073/pnas.200033797 (2000).
12. Han, X. *et al.* Reliability of mri-derived measurements of human cerebral cortical thickness: the effects of field strength, scanner upgrade and manufacturer. *NeuroImage* **32**, 180–94, DOI: 10.1016/j.neuroimage.2006.02.051 (2006).
13. Jovicich, J. *et al.* Reliability in multi-site structural mri studies: Effects of gradient non-linearity correction on phantom and human data. *NeuroImage* **30**, 436–443, DOI: 10.1016/j.neuroimage.2005.09.046 (2006).
14. Reuter, M., Rosas, H. D. & Fischl, B. Highly accurate inverse consistent registration: A robust approach. *NeuroImage* **53**, 1181–1196, DOI: 10.1016/j.neuroimage.2010.07.020 (2010).
15. Reuter, M., Schmansky, N. J., Rosas, H. D. & Fischl, B. Within-subject template estimation for unbiased longitudinal image analysis. *NeuroImage* **61**, 1402–1418, DOI: 10.1016/j.neuroimage.2012.02.084 (2012).
16. Desikan, R. S. *et al.* An automated labeling system for subdividing the human cerebral cortex on mri scans into gyral based regions of interest. *NeuroImage* **31**, 968–980, DOI: 10.1016/j.neuroimage.2006.01.021 (2006).

17. Jenkinson, M., Beckmann, C. F., Behrens, T. E., Woolrich, M. W. & Smith, M. A. Fsl. *NeuroImage* **62**, 782–790 (2012).

18. Tournier, J.-D. *et al.* Mrtrix3: A fast, flexible and open software framework for medical image processing and visualisation. *NeuroImage* **202**, 116137 (2019).

19. Veraart, J. *et al.* Denoising of diffusion mri using random matrix theory. *NeuroImage* **142**, 394–406 (2016).

20. Veraart, J., Fieremans, E. & Novikov, D. S. Diffusion mri noise mapping using random matrix theory. *Magn. Reson. Medicine* **76**, 1582–1593, DOI: 10.1002/mrm.26059 (2016).

21. Sairanen, V., Leemans, A. & Tax, C. M. W. Fast and accurate slicewise outlier detetion (solid) with informed model estimation for diffusion mri data. *NeuroImage* **181**, 331–346 (2018).

22. Andersson, J. L. R., Skare, S. & Ashburner, J. How to correct susceptibility distortions in spin-echo echo-planar images: application to diffusion tensor imaging. *NeuroImage* **20**, 870–888 (2003).

23. Smith, S. M. *et al.* Advances in functional and structural mr image analysis and implementation as fsl. *NeuroImage* **23**, S208–S219 (2004).

24. Andersson, J. L. R. & Sotiropoulos, S. N. An integrated approach to correction for off-resonance effects and subject movement in diffusion mr imaging. *NeuroImage* **125**, 1063–1078 (2016).

25. Kellner, E., Dhital, B., Kiselev, V. G. & Reisert, M. Gibbs-ringing artifact removal based on local subvoxel-shifts. *Magn. Reson. Medicine* **76**, 1574–1581 (2016).

26. Dhollander, T., Raffelt, D. & Connelly, A. Unsupervised 3-tissue response function estimation from single-shell or multi-shell diffusion mr data without a co-registered t1 image. *ISMRM Work. on Break. Barriers Diffusion MRI* **5** (2016).

27. Dhollander, T., Mito, R., Raffelt, D. & Connelly, A. Improved white matter response function estimation for 3-tissue constrained spherical deconvolution. *Proc Intl Soc Mag Reson. Med* 555 (2019).

28. Jeurissen, B., Tournier, J.-D., Dhollander, T., Connelly, A. & Sijbers, J. Multi-tissue constrained spehrical deconvolution for improved analysis of multi-shell diffusion mri data. *NeuroImage* **103**, 411–426 (2014).

29. Patenaude, B., Smith, S. M., Kennedy, D. N. & Jenkinson, M. A. A bayesian model of shape and appearance for subcortical brain segmentation. *NeuroImage* **56**, 907–922 (2011).

30. Smith, R. E., Tournier, J.-D., Calamante, F. & Connelly, A. Anatomically-constrained tractography: Improved diffusion mri streamlines tractography through effective use of anatomical information. *NeuroImage* **62**, 1924–1938 (2012).

31. Smith, S. M. Fast robust automated brain extraction. *Hum. Brain Mapp.* **17**, 143–155 (2002).

32. Zhang, Y., Brady, M. & Smith, S. Segmentation of brain mr images through a hidden markov random field model and the expectation-maximization algorithm. *IEEE Transactions on Med. Imaging* **20**, 45–57 (2001).

33. Jenkinson, M., Bannister, P. R., Brady, J. M. & Smith, S. M. Improved optimisation for the robust and accurate linear registration and motion correction of brain images. *NeuroImage* **17**, 825–841 (2002).

34. Jenkinson, M. & Smith, M. A. A global optimisation method for affine registration of brain images. *Med. Image Analysis* **5**, 143–156 (2001).

35. Woolrich, M. W. *et al.* Bayesian analysis of neuroimaging data in fsl. *NeuroImage* **45**, S173–S186 (2009).

36. Tournier, J.-D., Calamante, F. & Connelly, A. Improved probabilistic streamlines tractography by 2nd order integration over fibre orientation distributions. *Proc. Int. Soc. for Magn. Reson. Medicine* 1670 (2010).

37. Smith, R. E., Tournier, J.-D., Calamante, F. & Connelly, A. Sift2: Enabling dense quantitative assessment of brain white matter connectivity using streamlines tractography. *NeuroImage* **119**, 338–351 (2015).

38. Goñi, J. *et al.* Resting-brain functional connectivity predicted by analytic measures of network communication. *Proc. Natl. Acad. Sci.* **111**, 833–838, DOI: 10.1073/pnas.1315529111 (2014).

39. Honey, C. J. *et al.* Predicting human resting-state functional connectivity from structural connectivity. *Proc. Natl. Acad. Sci.* **106**, 2035–2040, DOI: 10.1073/pnas.0811168106 (2009).

40. Messaritaki, E. *et al.* Predicting meg resting-state functional connectivity from microstructural information. *Netw. Neurosci.* **5** (2021).

41. Mirza-Davies, A. *et al.* The impact of genetic risk for alzheimer's disease on the structural brain networks of young adults. *Front. Neurosci.* **16**, DOI: 10.3389/fnins.2022.987677 (2022).

42. Clarke, H. J., Messaritaki, E., Dimitriadis, S. I. & Metzler-Baddeley, C. Dementia risk factors modify hubs but leave other connectivity measures unchanged in asymptomatic individuals: a graph theoretical analysis. *Brain Connect.* **12**, 26–40 (2022).

43. Drakesmith, M. *et al.* Estimating axon conduction velocity in vivo from microstructural mri. *NeuroImage* **203**, 116186 (2019).

44. Messaritaki, E., Foley, S., Barawi, K., Ettinger, U. & Jones, D. K. Increased structural connectivity in high schizotypy. *Netw. Neurosci.* **7**, 213–233, DOI: 10.1162/netn_a_00279 (2023).

45. Assaf, Y. & Basser, P. J. Composite hindered and restricted model of diffusion (charmed) mr imaging of the human brain. *NeuroImage* **27**, 48–58, DOI: https://doi.org/10.1016/j.neuroimage.2005.03.042 (2005).

46. C, B. The biological basis of diffusion anisotropy. *Diffusion MRI* 155 – 183, DOI: https://doi.org/10.1016/B978-0-12-396460-1.00008-1 (2009).

47. Chen, Y. *et al.* White matter abnormalities revealed by diffusion tensor imaging in non-demented and demented hiv+ patients. *NeuroImage* **47**, 1154–1162 (2009).

48. Cykowski, M. D., Fox, P. T., Ingham, R. J., Ingham, J. C. & Robin, D. A. A study of the reproducibility and etiology of diffusion anisotropy differences in developmental stuttering: a potential role for impaired myelination. *NeuroImage* **52**, 1495–1504 (2010).

49. Jones, D. K., Knosche, T. R. & Turner, R. White matter integrity, fiber count and other fallacies: the do's and dont's of diffusion mri. *NeuroImage* **73**, 239–254 (2013).

50. Vrba, J. & Robinson, S. E. Signal processing in magnetoencephalography. *Methods* **25**, 249–271, DOI: 10.1006/meth.2001.1238 (2001).

51. Koelewijn, L. *et al.* Oscillatory hyperactivity and hyperconnectivity in young apoe-epsilon4 carriers and hypoconnectivity in alzheimer's disease. *eLife* **8** (2019).

52. Oostenveld, R., Fries, P., Maris, E. & Schoffelen, J.-M. Fieldtrip: Open source software for advanced analysis of meg, eeg, and invasive electrophysiological data. *Comput. Intell. Neurosci.* **2011**, 1–9, DOI: 10.1155/2011/156869 (2011).

53. Nolte, G. The magnetic lead field theorem in the quasi-static approximation and its use for magnetoencephalography forward calculation in realistic volume conductors. *Phys. Medicine Biol.* **48**, 3637–3652, DOI: 10.1088/0031-9155/48/22/002 (2003).

54. Hillebrand, A., Barnes, G. R., Bosboom, J. L., Berendse, H. W. & Stam, C. J. Frequency-dependent functional connectivity within resting-state networks: An atlas-based meg beamformer solution. *NeuroImage* **59**, 3909–3921, DOI: 10.1016/j.neuroimage.2011.11.005 (2012).

55. Colclough, G., Brookes, M., Smith, S. & Woolrich, M. A symmetric multivariate leakage correction for meg connectomes. *NeuroImage* **117**, 439–448, DOI: 10.1016/j.neuroimage.2015.03.071 (2015).

56. Colclough, G. *et al.* How reliable are meg resting-state connectivity metrics? *NeuroImage* **138**, 284–293, DOI: 10.1016/j.neuroimage.2016.05.070 (2016).

57. Godfrey, M. & Singh, K. D. Measuring robust functional connectivity from resting-state meg using amplitude and entropy correlation across frequency bands and temporal scales. *NeuroImage* **226**, 117551, DOI: 10.1016/j.neuroimage.2020.117551 (2021).
